# The Potential Impact of Blood System on Dietary Habits and Smoking

**DOI:** 10.3390/medicines9010003

**Published:** 2022-01-05

**Authors:** Ioannis Tsamesidis, Evangelia Stalika, Chinedu O. Egwu, Agathi Pritsa, Maria Parpori, Argyrios Gkinoudis, Diana Samara, Evgenia Lymperaki

**Affiliations:** 1 Department of Biomedical Sciences, International Hellenic University, 57001 Thessaloniki, Greece; evlimper@gmail.com; 2 School of Dentistry, Faculty of Health Sciences, Aristotle University of Thessaloniki, 54124 Thessaloniki, Greece; 3 Lab of Computing and Medical Informatics, Medical School, Aristotle University of Thessaloniki, 54124 Thessaloniki, Greece; evangelia.stalika@gmail.com; 4 PharmaDev, UMR 152, Université de Toulouse, IRD, UPS, 31000 Toulouse, France; echojay2010@yahoo.com; 5 Department of Nutritional Sciences and Dietetics, International Hellenic University, 57001 Thessaloniki, Greece; agpritsa@nutr.teithe.gr; 6 Department of Nursing, International Hellenic University, 57001 Thessaloniki, Greece; mariaprp@hotmail.com; 7 School of Veterinary Medicine, Aristotle University of Thessaloniki, 54124 Thessaloniki, Greece; ginoudisa@gmail.com; 8 Blood Bank Section, Naoussa General Hospital, 59200 Naousa, Greece; dianasamara7@gmail.com

**Keywords:** ABO blood groups, rhesus types, alcohol, caffeine, dietary habits, smoking

## Abstract

The ‘Blood-Type’ diet advises individuals to eat according to their ABO blood group to improve their health and decrease the risk of chronic diseases. However, the food preferences of individuals with different blood groups have not been examined. The aim of our study was to investigate, in healthy regular blood donors (rBDs), the associations of smoke, alcohol, caffeine, vitamin and fat intake with their different blood groups and if ABO groups could be a potential predictor tool for disease prevention. A total of 329 volunteers were divided into four groups according to their ABO types: Group 1 (A) comprised 141 rBDs; Group 2 (B), 65 rBDs; Group 3 (O), 96 rBDs; and Group 4, 27 rBDs. Additionally, they were divided into two groups according to their rhesus types and their preferences for smoke, too. Dietary intake was assessed using 3-day food recall and the Food Processor computer program for nutrient analysis. Alcohol, caffeine, sugar and Vitamin D consumption were significantly (*p* < 0.05) higher in the O group. The A group presented statistically significantly (*p* < 0.05) greater preferences for cholesterol intake and a higher trend for smoking (25%) habits compared with all the other groups, whereas Group B preferred more fatty foods. The blood group AB appeared to be the most controlled food intake group. Regarding the rhesus comparisons, alcohol; caffeine; and Vitamin C, D, E and K consumptions were significantly (*p* < 0.05) higher in rhesus-positive individuals than their rhesus-negative counterparts. For the non-smoker group, compared with the smokers, a higher consumption of Vitamin D and fibers was found. In conclusion, in the present study, statistically significant correlations of the ABO and rhesus system with some dietary parameters were found, indicating a consequent influence of these preferences on the progression of different diseases.

## 1. Introduction

ABO blood grouping is one of the first genetic variations recognized in humans and has since been linked to several health conditions. Since the publication of the book “*Eat Right for Your Type”* by D’Adamo [1], there has been a series of debates for and against the correlation of an individual’s ABO blood group, eating habits and health. This theory considers our ancestral dietary habits and suggested that adherence to diets specific to one’s blood type can reduce the risk of cardiovascular disease and improve health generally [1]. The most common food preferences comprise caffeine, alcohol, fruits, and vegetables containing vitamins and fats. Caffeine is a nutrient that can be found in many beverages and plays an important role in health because of its interference in Vitamin B and essential mineral absorption [2]; moreover, in low doses, besides producing an increase in mental energy and attention, and elevated mood, it also has antioxidant properties [3]. Vitamins, and especially Vitamins A and D, have a crucial effect on the regulation of immune responses and have a protective role in inflammation and autoimmunity. They participate in the production of specific antibodies, the proliferation of lymphocytes, and T cell differentiation [4].

Wang et al. demonstrated that, although adhering to some blood-type diets may reduce cardiometabolic risk factors, these links are independent of an individual’s ABO group [5]. On the other hand, research on the ABO group system proposed an association between ABO blood groups and a variety of diseases such as hepatitis [6], thrombotic disorders [7], metabolic and cardiovascular diseases [8,9], cognitive disorders and circulatory diseases. Moreover, AB individuals were found to be linked with an increased incidence of smallpox, E. coli and Salmonella infections [10]. Additionally, the blood type O presents a connection with an increased incidence of cholera, plague and tuberculosis infections, whereas the blood type A seems to be linked with an increased incidence of Pseudomonas aeruginosa infection [10]. Additionally, the ABO blood system has been verified to be linked with the severity of some infectious diseases such as malaria for the B group [11,12,13] and COVID-19 for the A group [14,15,16]. In the literature, a few studies have demonstrated the analysis of dietary habits as well as smoking to investigate the possible association with the blood group system. In the past, studies to demonstrate the association of smoking with ABO blood groups were conducted, without significant results [17,18,19]. In a cohort study, Jaleel et al. demonstrated that people having tobacco-chewing habits with blood group A were at a 1.46-times greater risk of developing oral cancer, compared with those of blood groups B, AB and O [17]. Regarding eating habits, according to Leite et al., one’s ABO blood group affects one’s tastes and preferences [20]. The blood group antigens are differentially expressed in the sensory cells of the auditory, taste and olfactory systems. The results of a cohort study demonstrated that the ability to recognize phenylthiocarbamide (PTC) in a taste test is related to blood group B and a high risk of developing food allergies; however there is no consensus on whether this actually affects taste in individuals with different ABO groups [21,22,23]. Additionally, there are a few reports of the association of the O blood group and addiction to alcohol and opioids [24,25]; however, there is no consensus on the exact correlation between ABO groups and addiction/preferences. Moreover, Raman et al. demonstrated the effect of plant lectins on human blood group binding activity, indicating that the O blood group showed the most significant activity compared with other blood groups [26]. The growing association of diseases with ABO blood groups and the paucity of evidence linking ABO groups and tastes/preferences call for an intensified effort in understanding the correlation between diet and health conditions in individuals with varying ABO statuses. This work, therefore, aimed to investigate the associations and trends of the smoke, alcohol, caffeine, vitamin and fat intake of healthy regular blood donors (rBDs) with their blood groups. 

## 2. Materials and Methods 

### 2.1. Study Design

A pilot study was carried out at the Blood Bank of Naousa Hospital, Greece. The blood donors (n = 329) comprised 182 males aged 19–61 years and 147 females aged 21–64 years. All the participants provided written informed consent before the study and were asked to fill out a short questionnaire about sociodemographic characteristics such as the age, sex, body mass index (BMI), smoking habits and residence of the blood donors. All the volunteer regular blood donors (rBDs) were selected, recognizing them as a healthy population, excluding drug and supplement intake.

### 2.2. Dietary Intake (DI) Assessment

A 3-day food recall (one weekend day and two weekdays) was used to estimate the dietary intake. All the participants were asked to describe, in detail, the quantity and type of food consumed during those 3 days. The 3-day dietary intake recalls were conducted in face-to face interviews by experienced nurses and dietitians of the hospital. Written and verbal instructions were provided in order to complete their food recalls. Dietary intake was assessed using the Food Processor computer program for nutrient analysis (ESHA, Salem, OR, USA, 2010) (version 7.4) with Greek foods, as previously performed [27]. The study mainly used the Greek food composition in the EuroFIR AISBL e-book collection by the Hellenic Health Foundation to convert dietary data to nutrient intakes [28].

### 2.3. AΒO Blood and Rhesus System Detection

Just before blood donation, the A, B, O and AB groups and rhesus statuses were obtained for all the participants from the Blood Bank in General Hospital in Naousa using the Ortho Biovue clinical diagnostics system.

### 2.4. Ethical Statement 

The study was conducted in accordance with Good Clinical Practice guidelines and the Declaration of Helsinki. Ethical approval to perform the present study was obtained from the Ethical Committee of the General Hospital of Naousa (ID_233205920). The confidentiality of the participants was wholly preserved. 

### 2.5. Statistical Analysis

To compare biochemical markers with the ABO blood group and rhesus type, statistical analysis was performed using the SPSS tool version 22.0. Descriptive statistics, presented as the mean ± standard deviation, were performed. Additionally, inferential statistical analysis (*t*-test) was used for investigating the possible differences between two blood groups regarding the biochemical markers’ statuses. One-way ANOVA tests further evaluated possible differences in the lipidemic and anemic predispositions among the blood groups. In all the statistical analysis, the level of significance (*p*-value) was set at α = 0.05. The independent variables with *p* < 0.05 in bivariate correlation analysis were enrolled in binary logistic regression for further analysis. A logistic regression model was used to examine the effect of particular independent variables on different ABO blood groups.

## 3. Results and Discussion

The distributions of the blood groups across the different subpopulations in our study are presented in Table 1. The study population was selected based on the inclusion criteria outlined in the methodology. Demographic analysis of the 329 donors showed 60%, 45%, 50% and 50% males for A, B, O and AB, respectively. The ABO blood group frequencies in the Greek population revealed A as the most frequent, followed by O, B and AB, respectively, in accordance with other regional studies [29,30]. The BMI levels of all the rBDs did not present any significant differences.

Correlations between ABO blood group smoking and dietary intakes in study population are presented in Table 2. For the entire study population, alcohol, caffeine and sugar consumption were significantly (*p* < 0.05) higher in the O group. In addition to several intervening factors, the taste of alcohol and other beverages may also influence the highest consumption of blood group O without necessarily promoting disease generation [31]. Moreover, Vitamin D consumption was higher, too, confirming its relationship with COVID-19 [32] and their in-between correlation [15]. The A group presented significantly (*p* < 0.05) greater preferences for cholesterol and fiber intake and a higher trend for smoking (25%) habits compared with all the other groups. This fact is in accordance with other studies [8,33] related to the association of the A blood type with a high risk of cardiovascular diseases. On the other hand, the intakes of vitamins, such as Vitamins A and C, was higher in blood group A, indicating their health benefits in developing a dietary balance. All the rBDs followed a traditional Mediterranean diet based on the Greek food pyramid guidelines. The food pyramid is divided into three levels of consumption: the daily consumption of wholegrain cereals and products, fruits, vegetables and olive oil; secondly, the weekly consumption of fish, poultry, olives, pulses, nuts, potatoes, eggs and sweets; and monthly, red meat. 

Nordmo demonstrated a significant association between blood group A and alcoholism; however, this claim may have been limited by the shortcomings of the research, which included sampling errors [34,35]. Meanwhile, those with blood group B preferred more fatty foods, confirming the observations of other investigations indicating that the rate of hypertension for the blood type B is the maximum, compared with the other blood groups [36,37]. The blood group AB appears to show the most controlled food intake overall, indicating that the blood type AB is protective for hyperlipidemia and other common diseases, too. 

The impact of rhesus factors on the consumption of different diets is presented in Table 3. For the study population, the alcohol; caffeine; and Vitamin C, D, E and K consumptions were significantly (*p* < 0.05) higher in rhesus-positive individuals than their rhesus-negative counterparts. Cholesterol consumption was also significantly higher among rhesus-positive individuals than their rhesus-negative counterparts. Rhesus factor status did not significantly (*p* > 0.05) affect the consumption of Vitamin A, Ω3, Ω6, fiber and sugar. In the same vein, there was no statistical (*p* > 0.05) difference in the consumption of fat and different types of fat (sat.fat, MUFA and PUFA) between rhesus-positive and rhesus-negative individuals in the population studied. Rhesus factor is a type of protein present on the surface of red blood cells, and its presence (rhesus positive) or absence (rhesus negative) can trigger different types of reactions to different diets. Rhesus-negative individuals are prone to more immunoglobulin E allergies than rhesus-positive individuals [38]. Different diets trigger IgE differently. The reported differences in responses could be due to the differences in IgE triggers in these individuals with either rhesus-positive or negative status. The fear of allergic reactions, which could be life threatening in some cases, may influence the choice of a particular type of food. 

Smokers are an important target group for dietary intervention, and their preferences should be evaluated in order to analyze their eating patterns for this reason; the preferences of the smokers and non-smokers in the study population are presented in Table 4. There was no in-between group difference in the consumption of alcohol; caffeine; cholesterol; and Vitamins C, E, K and A; however, for Vitamin D, the consumption was significantly higher in non-smokers than in smokers. The consumption of Ω3 was also similar in both groups, while for Ω6 and sugar, it was higher in non-smokers. For fiber, non-smokers showed statistically significant greater preferences compared with the smokers (*p* = 0.001). The consumption of fat and the different types of fat (saturated fat and PUFA) were generally similar in both groups, except for MUFA, known as the healthier fat, which was significantly higher in non-smokers. Our data and previous analysis [39,40] in smokers and non-smokers revealed that the non-smokers were following healthier dietary consumptions compared with the smokers. Although there were no significant in-between group differences for most diets (except for Ω6, sugar, fiber, MUFA and Vitamin D), it has been previously reported that cigarette smokers have more difficulties in controlling different cravings [41].

## 4. Conclusions

Previous reports have provided some evidence for associations between dietary intakes and the ABO blood system. In the present population, we found statistically significant correlations of the ABO and rhesus system with some dietary parameters, which are also associated with certain diseases and their in-between correlations with the blood system. Food preferences can influence the risks of different diseases without certainly being the exact cause of diseases. Moreover, the health community and the medical nutritionists should take into consideration the blood system as an important factor before planning a diet. Further studies in a larger population and in different ethnicities will provide more insights into the role of the blood system in diet and disease progression. 

## Figures and Tables

**Table 1:** Characteristics of all study participants.

Blood Groups (*N* = 329)				
	A (*N* = 141) Median ( ±SD)	B (*N* = 65) Median ( ±SD)	O (*N* = 96) Median ( ±SD)	Ab (*N* = 27) Median ( ±SD)
**Sex, Male (%)**	60	45	50	50
**Age**	42 (±12.08)	43 (±9.55)	46 (±9.26)	44 (±8.54)
**BMI**	26.8 (±2.51)	26.3 (±2.15)	26.4 (±1.87)	26.2 (±1.90)

**Table 2:** Correlation between ABO blood group smoking and dietary intakes in study population. Statistically significant differences are presented with *.

Blood Types					
	A	B	O	AB	
Parameters	Average Values				*p-*Value
Alcohol (g)	0.86	1.49	8.90	1.55	0.001 *
Caffeine (mg)	95.12	241.26	262.21	115	0.001 *
Smoking (%)	25	21	12	20	0.05 *
Vitamin C (mg)	159.63	62.44	116.56	65.74	0.015 *
Vitamin D (μg)	1.91	1.89	3.79	1.52	0.001 *
Vitamin E (U)	4.49	5.25	6.59	3.74	0.004 *
Vitamin K (μg)	24.64	18.07	15.16	16.83	0.359
Vitamin A (IU)	3925	3171	3045	3101	0.061
Cholesterol (mg)	251.34	197.2	126.2	188.2	0.050 *
Ω3 (g)	0.70	0.66	0.55	0.71	0.799
Ω6 (g)	6.25	5.43	4.87	7.01	0.329
Fat (g)	49.08	63.12	56.28	55.12	0.050 *
Sugar (g)	43.39	42.69	52.02	35.25	0.188
Fiber (g)	28.83	11.02	22	13.23	0.05
Sat. Fat (g)	20.25	21.39	20.85	21.28	0.857
MUFA (g)	19.70	21.18	23.15	15.96	0.020 *
PUFA (g)	9.41	9.13	9.95	7.64	0.677

**Table 3:** Correlation between rhesus types and preferences in study population. Statistically significant differences are presented with *.

Rhesus Types			
Parameters	Positive *(N* = 269*)*	Negative *(N* = 60*)*	*p*-Value
Alcohol (g)	3.508	2.880	0.03 *
Caffeine (mg)	187.91	119.88	0.01 *
Smoking (%)	26	33	0.05 *
Vitamin C (mg)	106.12	183.07	0.05 *
Vitamin D (μg)	4.33	2.08	0.02 *
Vitamin E (U)	5.43	4.14	0.02 *
Vitamin K (μg)	21.10	14.68	0.03 *
Vitamin A (IU)	3534	3080	0.19
Cholesterol (mg)	243.7	198.2	0.03 *
Ω3 (g)	0.676	0.658	0.36
Ω6 (g)	5.88	5.17	0.31
Fat (g)	54.80	48.11	0.15
Sugar (g)	45.68	42.52	0.22
Fiber (g)	23.62	14.96	0.13
Sat. Fat (g)	21.05	18.63	0.25
MUFA (g)	21.40	17.49	0.07
PUFA (g)	9.60	7.96	0.15

**Table 4:** Correlation of smokers and non-smokers with preferences in study population. Statistically significant differences are presented with *.

Blood Donors				
Parameters	Smokers *(N* = 84*)*	Non-Smokers *(N* = 245*)*	*p*-Value	Pearson’s *R*
Alcohol (g)	3.23	3.44	0.97	0.13
Caffeine (mg)	182.90	171.49	0.22	0.09
Vitamin C (mg)	128.69	117.23	0.15	0.11
Vitamin D (μg)	1.38	4.79	0.04 *	0.46
Vitamin E (U)	4.97	5.27	0.26	0.12
Vitamin K (μg)	24.67	18.31	0.006 *	0.38
Vitamin A (IU)	3741	3352	0.09 *	0.16
Cholesterol (mg)	179.3	184.5	0.75	0.22
Ω3 (g)	0.80	0.63	0.23	0.27
Ω6 (g)	5.04	5.99	0.02 *	0.31
Fat (g)	45.29	56.42	0.7	0.44
Sugar (g)	41.76	46.25	0.009 *	0.24
Fiber (g)	13.16	25.08	0.001 *	0.25
Sat. Fat (g)	18.88	21.20	0.14	0.19
MUFA (g)	18.25	21.53	0.019 *	0.31
PUFA (g)	8.22	9.65	0.11	0.16

## Data Availability

Data is contained within the article.
